# Visualization of drug delivery processes using AIEgens

**DOI:** 10.1039/c6sc05421h

**Published:** 2017-01-18

**Authors:** Youyong Yuan, Bin Liu

**Affiliations:** a Department of Chemical and Biomolecular Engineering , National University of Singapore , 4 Engineering Drive 4 , Singapore 117585 . Email: cheliub@nus.edu.sg; b Institute of Materials Research and Engineering , Agency for Science , Technology and Research (A*STAR) , 3 Research Link , Singapore 117602

## Abstract

Drug delivery systems (DDSs) have been extensively studied as carriers to deliver small molecule chemo-drugs to tumors for cancer therapy. The therapeutic efficiency of chemo-drugs is crucially dependent on the effective drug concentrations in tumors and cancer cells. Novel DDSs that can simultaneously unveil drug distribution, drug release/activation behaviors and offer early evaluation of their therapeutic responses are highly desirable. Traditional fluorescent dye-labeled DDSs may suffer from notorious aggregation-caused quenching (ACQ) with limited sensitivity for bioimaging; in addition, the intrinsic fluorescence of these dyes requires careful selection of energy acceptor or quencher moieties for a light-up probe design, which complicates the development of self-reporting DDSs, especially the ones for reporting multiple processes. The recently emerged fluorogens with aggregation-induced emission characteristics (AIEgens) offer a straightforward solution to tackle this challenge. Thanks to the unique properties of AIEgens, new theranostic DDSs have been developed for simultaneous drug delivery and bioimaging with high signal to background ratio and multiple signal reporting capabilities. In this mini-review, we summarize the recent development of theranostic DDSs based on AIEgens for monitoring the drug distribution, drug activation and prediction of the therapeutic responses. Through illustration of their design principles and application examples, we hope to stimulate the interest in the design of more advanced theranostic DDSs for biomedical research.

## Abstract 2

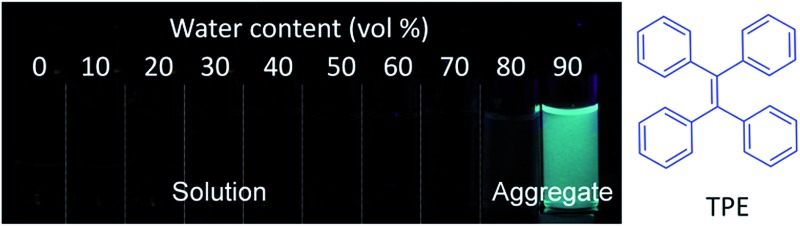
We summarize the recent development of AIEgen based theranostic DDSs for the visualization of drug delivery processes.

## Introduction

Drug delivery systems (DDSs) have been extensively studied as carriers to deliver small molecule chemo-drugs to tumors for cancer therapy.^1^ The therapeutic efficiency of chemo-drugs is crucially dependent on the effective drug concentrations in tumors and cancer cells. Upon intravenous injection of the pharmaceutically active payload, the drug will accumulate in the tumor tissue through passive or active targeting, then pass through the tumor extracellular matrix (ECM),^2^ bind to cells and cross the cell membrane to enter the targeted cancer cells.^3,4^ After being taken-up by cancer cells, the drug release or activation rate is crucial for therapeutic outcome. Similarly, prediction of the drug therapeutic effect is of equal importance to evaluate whether the therapeutic regimes work well, which is of great value to guide the therapeutic decisions made by doctors. Therefore, it is important to develop novel theranostic DDSs that can unveil their drug distribution, offer accurate assessment of drug release or activation in cancer cells and predict their therapeutic responses in cancer therapy. This will provide important information to further optimize the therapeutic regimes in order to advance the therapy. However, as most of the anticancer drugs are non-emissive, our capability remains limited in precisely answering when, where and how the anticancer drugs are delivered to cells. The tasks of accurate assessment of drug release or activation after cellular uptake as well as early evaluation of their therapeutic responses are even more challenging. However, the strong interest in personalized medicine calls for the development of theranostic DDSs that could provide a clear answer to all of these questions.^5^ So far, fluorescent dyes have been widely used for labeling DDSs to study their biodistribution.^6,7^ After their accumulation in tumor sites and being taken-up by cancer cells, the drug activation process was monitored *in vitro* and *in vivo*, particularly for anticancer drugs conjugated with fluorogenic dyes through tumor microenvironment responsive linkers.^8–10^ Recently, real-time monitoring of the therapeutic responses has been realized by imaging the drug induced cancer cell apoptosis, a pathway commonly involved in cell death.^11,12^ The majority of theranostic DDSs relies on fluorescent dyes with aggregation-caused quenching (ACQ) to report the signal, which limits the detection sensitivity. In addition, the intrinsic fluorescence of most dyes requires careful selection of energy acceptors or quencher moieties to realize fluorescence change, which complicates the design of self-reporting DDSs, especially the ones for reporting multiple processes.

**Fig. 1:**
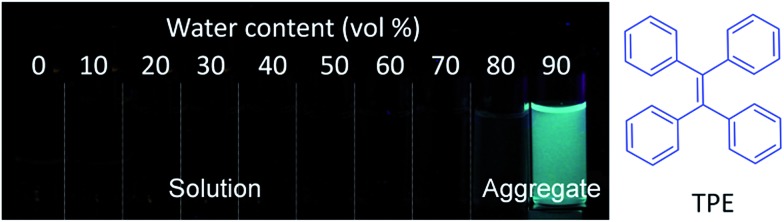
Fluorescence photographs of solutions or aggregates of tetraphenylethene (TPE) in THF/water mixtures with different amounts of water (vol%), showing a typical AIE phenomenon.

The discovery of a novel class of fluorogens with aggregation-induced emission characteristics (AIEgens) has recently received intense research interest in biosensing and bioimaging because AIEgens offer a straightforward solution to the ACQ problem.^13,14^ Opposite to the photophysical phenomenon of ACQ dyes, AIEgens are almost non-emissive in the molecularly dissolved state but emit intensely in the aggregated state due to the restriction of intramolecular motions and prohibition of energy dissipation *via* non-radiative decay channels.^15^ Tetraphenylethene (TPE), an iconic AIEgen, is of particular research interest. The central olefin stator of the TPE molecule is surrounded by four phenyl rings. As shown in Fig. 1, the TPE molecules in a benign solvent are non-emissive, which is attributed to dynamic rotations of the four phenyl rings non-radiatively dissipating exciton energy. In the aggregated state, the emission of TPE is induced or rejuvenated by the synergistic effects of the restriction of intramolecular rotation (RIR) and the highly twisted molecular conformation that hampers the intermolecular π–π stacking interaction.^14^ The unique features of various AIEgens with tunable absorption and emission colors have led to the development of simple light-up probes^16,17^ and very bright AIE nanoparticles,^18–20^ making them promising tools for biomedical applications.

**Fig. 2:**
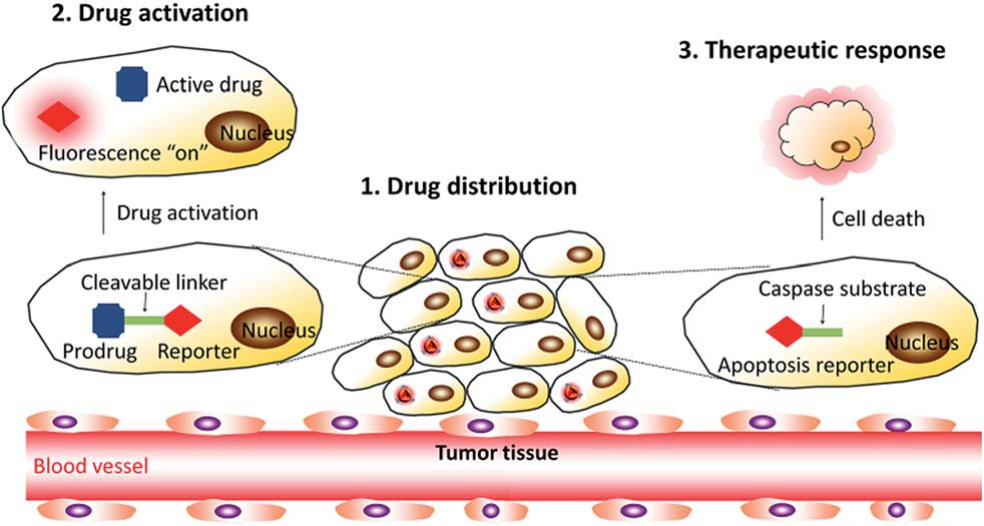
Schematic representation of the DDSs based on AIEgens used for monitoring the drug distribution, drug activation, and *in situ* prediction of therapeutic responses.

In this review, we summarize the recent development of AIEgen based theranostic DDSs for the visualization of drug delivery processes (Fig. 2). The review is organized according to the three steps of the drug delivery process that theranostic DDSs are monitored by, starting from drug distribution, which is followed by drug activation and evaluation of therapeutic responses. In each subsection, the design principle of the corresponding theranostic DDSs is discussed, which is further elaborated by examples of specific imaging and therapy applications. Finally, perspectives for the future development of theranostic DDSs are discussed.

## Drug distribution monitoring

To monitor the drug distribution of DDSs upon intravenous injection and internalization into selected cancer cells, the commonly used strategy is to label the DDSs through encapsulation/conjugation with fluorescent dyes or quantum dots.^21^ However, these methods may suffer from some problems, such as fluorescence quenching associated poor imaging sensitivity of fluorescent dyes^22^ or the potential toxicity of quantum dots.^23^ Therefore, it is highly desirable to develop biocompatible self-luminescent DDSs for real-time monitoring of the drug distribution upon intravenous injection. Inspired by the unique properties of AIEgens that are highly fluorescent in the aggregated state, several AIEgen based theranostic DDSs have been developed recently to monitor the drug distribution with promising results.

**Fig. 3:**
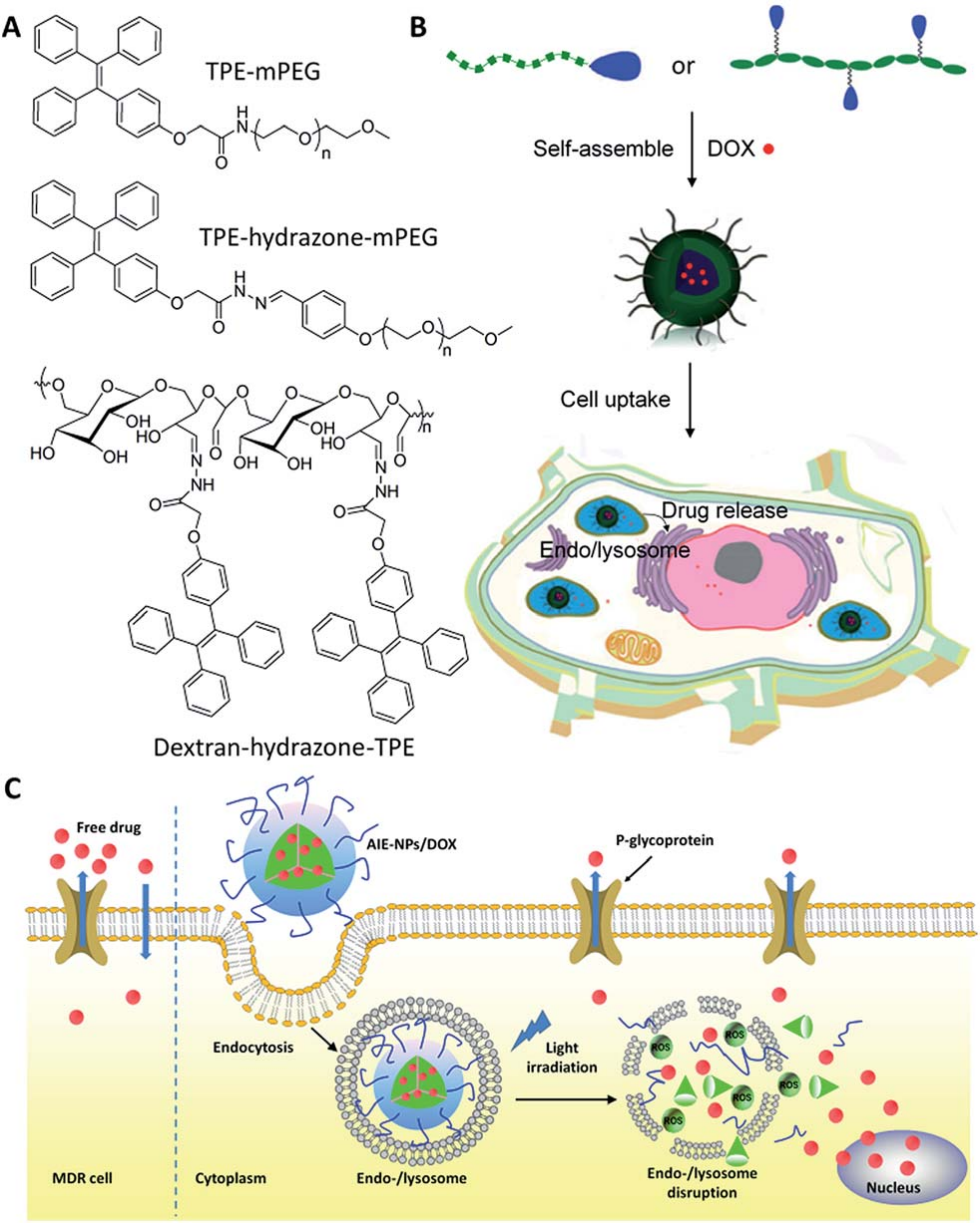
Schematic illustration of drug-loaded micelles with AIE properties as novel theranostic platforms for intracellular imaging and cancer treatment. Reprinted with permission from ref. 24, 26 and 27. Copyright 2014 American Chemical Society. Copyright 2015 Royal Society of Chemistry.

To construct theranostic DDSs for simultaneous cancer cell imaging and drug delivery, Liang *et al.* developed a traceable DDS using TPE derivatives to fabricate self-assembled micelles to visualize the intracellular drug distribution.^24^ In this work, a hydrophilic and biocompatible polymer, polyethylene glycol (PEG), is conjugated to TPE to form an amphiphilic AIE polymer (TPE–mPEG), which can self-assemble into micelles in aqueous solution and allow efficient loading of anticancer drug doxorubicin (DOX) (Fig. 3). The critical micelle formation concentration (CMC) of the AIE polymer was determined by the fluorescence intensity change of TPE.^25^ The DOX loaded AIE micelle shows blue fluorescence, which has been used to track the intracellular drug distribution at the subcellular level. The DDS enabled a drug loading content as much as 15.3% by weight, and the anticancer efficiency was higher than that using free DOX.

Later on, Wang *et al.* developed two pH-responsive amphiphilic polymers based on TPE to monitor the intracellular distribution and drug release (Fig. 3).^26,27^ These amphiphilic polymers (TPE–hydrazone–mPEG and dextran–hydrazone–mPEG) self-assembled into micelles with the encapsulation of DOX, which exhibited blue fluorescence of TPE in aqueous media. The DDSs were located in endo/lysosomes after cellular uptake, which were revealed by the colocalization of the blue fluorescence of TPE and lysotracker green as an endo/lysosome specific fluorescent tracker. Moreover, the DOX showed pH-regulated release behavior and the released drug in acidic endo/lysosomal could be imaged by the fluorescence increase of TPE due to the cleavage of the hydrazone bond, which terminated the energy transfer between TPE and DOX to recover the quenched TPE fluorescence. In addition, *in vitro* results demonstrated that both of the drug-loaded nanoparticles exhibited dose-dependent cytotoxicity to HeLa cells. Recently, Liu *et al.* also developed a light-responsive amphiphilic AIE polymer for drug delivery (AIE-NPs/DOX) and imaging the cytosolic drug release to overcome drug resistance in cancer cells (Fig. 3C).^28^ In this work, the distribution of the drug was monitored by tracking the red fluorescence of the AIE molecules, which revealed the endo/lysosome trapping of DOX and subsequent drug release to the cell cytoplasm. This design strategy can significantly improve the intracellular DOX accumulation and retention in DOX resistant MDA-MB-231 cells to significantly inhibit cancer cell growth. The half maximal inhibitory concentration (IC_50_) of AIE-NPs/DOX with light irradiation is ∼12% of free DOX and ∼32% under dark conditions after 72 h incubation.

**Fig. 4:**
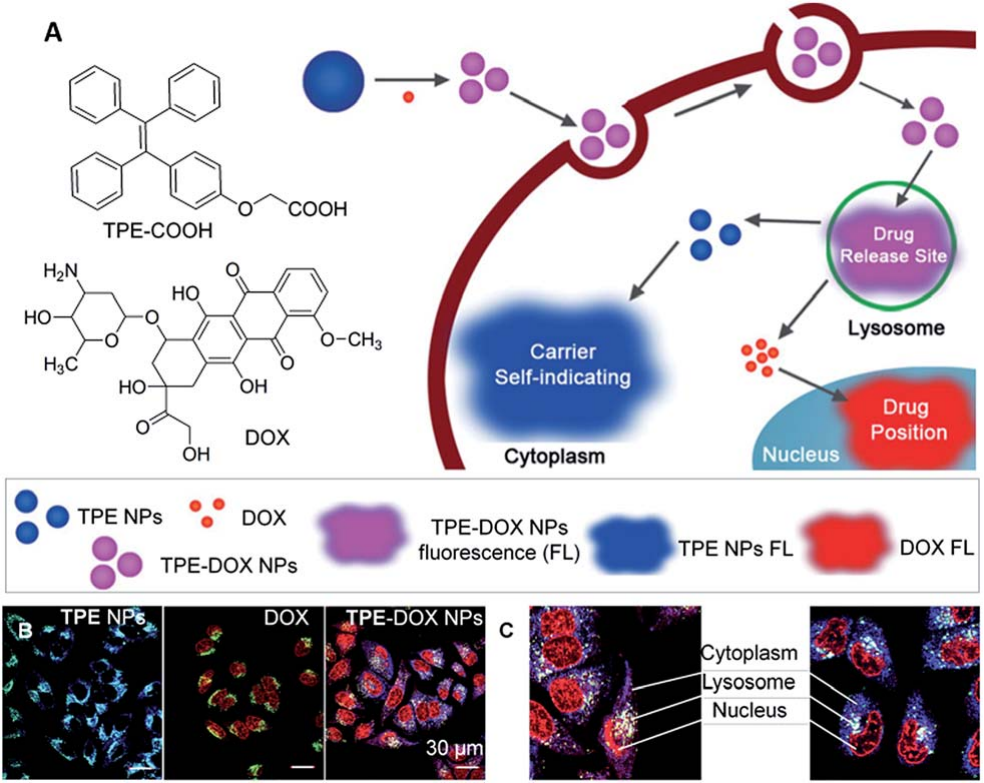
(A) Schematic illustration showing the formation of TPE–DOX NPs, the intracellular drug releasing site, and real time sub-cellular imaging of TPE NPs and DOX by emission color transitions. (B) Spatial distributions of TPE NPs, DOX and TPE–DOX NPs in MCF-7s cells. CLSM images of TPE NPs, DOX and TPE–DOX NPs distribution; lysosomes were stained by Lysotracker Green. (C) Detailed TPE–DOX NPs spatiotemporal distribution in MCF-7s cells. Reprinted with permission from ref. 29. Copyright 2014 Wiley-VCH.

Soon afterwards, Liang *et al.* developed a self-indicating DDS based on fluorescence resonance energy transfer (FRET) between TPE and DOX for spatiotemporal visualization of the drug distribution and release.^29^ The self-luminescent TPE nanoparticles (TPE NPs) can easily bond with DOX to form TPE–DOX NPs *via* electrostatic interactions in aqueous solution (Fig. 4A). TPE NPs with a blue color showed no obvious cytotoxicity and were distributed in the cytoplasm rather than entering the nuclei of cells where DOX functions. Due to the protonation of DOX at low pH, the release of DOX from TPE–DOX NPs was accelerated at pH 5, causing the DOX to be released in lysosomes. As can be seen from the fluorescence images shown in Fig. 4B, TPE–DOX NPs, TPE NPs and free DOX displayed three distinct “colors” of purple, blue and red, respectively. The subcellular distribution and the drug release of the DDSs can be determined by observing the transition of those “colors”. Specifically, after cellular uptake, TPE–DOX NPs were first entrapped in the lysosomes, showing a purple color; once the DOX was released, the separation between the TPE-COOH and DOX caused the color to change, *i.e.* the released DOX could diffuse to the cell nuclei and light up with a red color, while the TPE-NPs staying in the cytoplasm showed a blue color (Fig. 4C). As such, the theranostic DDS clearly provided the sub-cellular information of the drug distribution by offering distinct color changes. The TPE–DOX NPs are 2-fold more effective in inhibiting the proliferation of cancer cells than free DOX.

**Fig. 5:**
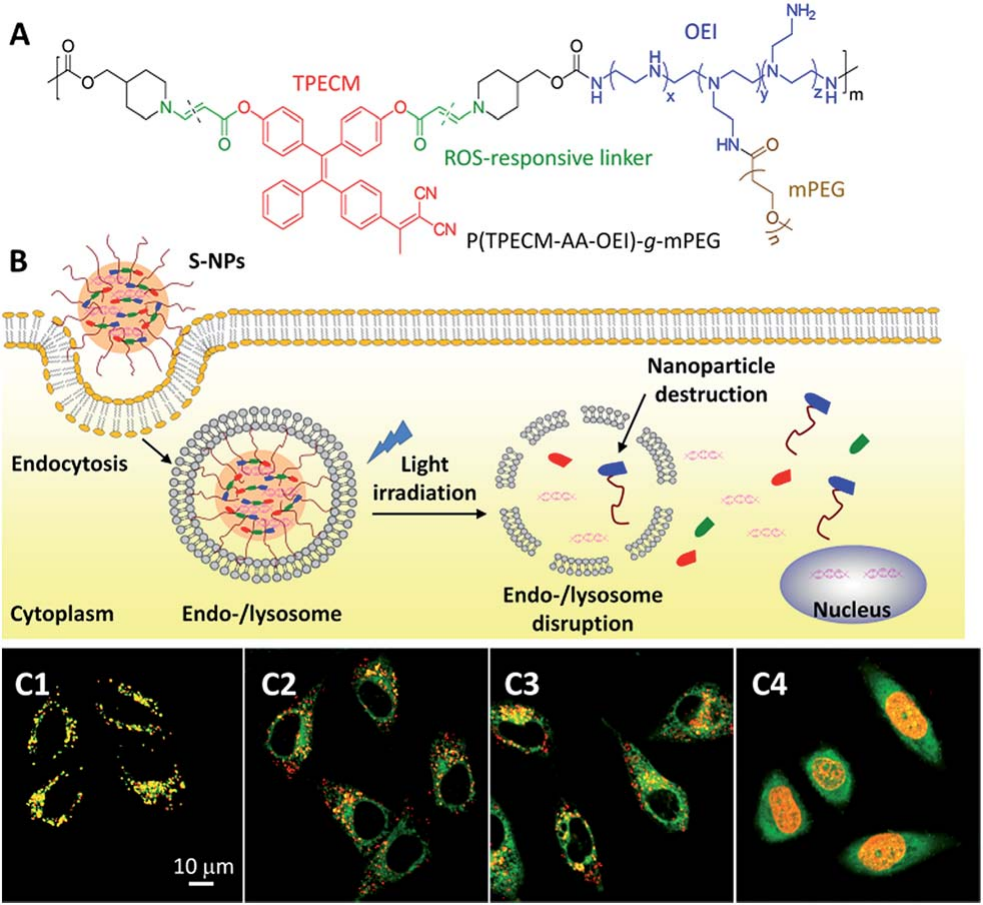
(A) Chemical structure of a reactive oxygen species (ROS) responsive polymer. (B) The mechanism of ROS responsive nanoparticles (S-NPs) complexed with DNA (S-NPs/DNA) to the transgene expression. (C) CLSM images of HeLa cells incubated with YOYO-1 labeled S-NPs/DNA complexes (C1) in the dark, and with light irradiation for (C2) 2 min and (C3) 5 min. Green: YOYO-1 fluorescence (*E*_x_: 488 nm; *E*_m_: 505–525 nm); red: S-NPs fluorescence (*E*_x_: 405 nm; *E*_m_: > 560 nm). Yellow: co-localization of red and green pixels. (C4) CLSM image illustrating the localization of YOYO-1–DNA after light irradiation with a further 4 h incubation. Green: YOYO-1 fluorescence; red: living nuclei stained with DRAQ5 (*E*_x_: 633 nm; *E*_m_: > 650 nm); yellow: co-localization of red and green pixels. Reprinted with permission from ref. 30. Copyright 2015 Wiley-VCH.

In addition to drug delivery, AIEgens have also been applied for monitoring the intracellular distribution of gene delivery. For gene therapy, the encapsulated nucleic acids need to escape from the enzyme-rich endo-/lysosome, resulting in release into the cytosols to reach their final destination in the nucleus. It is thus important to visualize the location of genes at different time scales. Liu *et al.* developed reactive oxygen species (ROS) sensitive nanoparticles (S-NPs) for light controlled gene delivery (Fig. 5A).^30^ This polymer consists of oligoethylenimine (OEI) and an AIE photosensitizer (TPECM) conjugated through a ROS reactive aminoacrylate linker. The polymer can self-assemble into S-NPs and bind with DNA to show bright red fluorescence. Once the DNA is intercalated with YOYO-1, the S-NPs/DNA complexes have a yellow emission, which can be used for tracking the gene delivery. After cellular uptake, yellow fluorescence was observed in the endo/lysosomes, indicating the initial location of the genes in the cells (Fig. 5C1). Under white light illumination, the generated ROS from TPECM can concurrently induce DNA unpacking and endo-/lysosomal escape. This was visualized by the separation between the red fluorescence from the S-NPs and the green fluorescence from the DNA/YOYO-1 (Fig. 5C2 and C3), indicating that the DNA was released from the S-NPs. After additional incubation of the cells for 4 h in cell culture medium, the green fluorescence of YOYO-1 was found in the nucleus (Fig. 5C4), which co-localized well with DRAQ-5 dye, a commercial near infrared (NIR) fluorescent nucleus stain to yield yellow fluorescence. The successful gene delivery led to over 50% increase in gene transfection efficiency as compared with the gold standard polyethylenimine (PEI, average molecular weight of 25k). Once the nucleic acids are substituted with therapeutic DNA or siRNA,^31^ this simple yet effective gene delivery system has the potential to be applied to efficient gene therapy.

## Drug activation monitoring

An effective theranostic DDS should improve the therapeutic indices and also minimize the side effects of drugs. The currently clinical chemotherapeutic drugs often suffer from severe side effects, which hampered their effectiveness in cancer therapy. To address these limitations, non-toxic prodrugs, which can restore their latent cytotoxic activity in the tumor microenvironment, have been developed.^32^ Tumor microenvironment induced drug activation for selective cancer cell ablation is highly desirable to minimize the side effects to normal cells. It has always been of interest to develop a theranostic prodrug delivery system that can simultaneously deliver the prodrugs to cancer cells and meanwhile offer real-time monitoring of the prodrug activation *in situ* to provide quantitative readout of active drug concentration in living cells. To achieve this goal, the drugs are usually conjugated with fluorogenic dyes through a tumor microenvironment responsive linker.^8^ The fluorescence change can be synchronously observed upon the drug activation, giving a quantitative readout of active drugs. However, most of the traditional dyes are not fluorogenic and the incorporation of energy accepting quenchers complicates the DDSs. Recently, several theranostic DDSs based on AIEgens with simple designs have been developed for real-time monitoring of the drug activation in cancer cells.

**Fig. 6:**
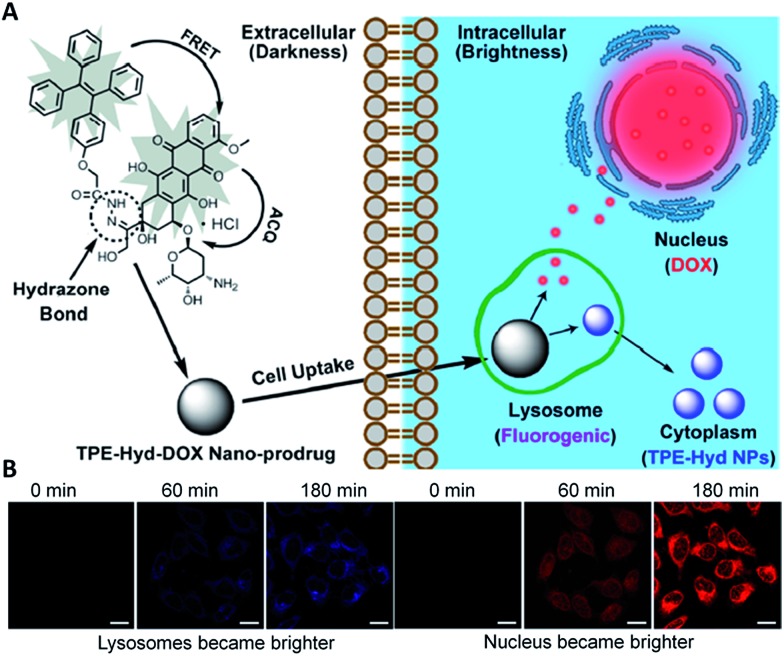
Intracellular trajectory of nanoscaled drug delivery systems of the TPE–Hyd–DOX nano-prodrug in cells. Reprinted with permission from ref. 33. Copyright 2015 American Chemical Society.

Recently, Liang *et al.* developed a traceable prodrug to track drug delivery kinetics in living cells.^33^ The self-indicating prodrug TPE–Hyd–DOX constitutes two fluorophore moieties, TPE and a fluorescent anticancer drug DOX, which are conjugated through a pH-responsive hydrazone bond (Fig. 6A). Under physiological conditions, and upon excitation of the energy donor TPE at 330 nm, the fluorescence of both TPE and DOX is quenched. The former is due to the energy transfer between TPE and DOX, and the latter is due to self-quenching effect of DOX. Under acidic conditions, the cleavage of the hydrazone bond separated TPE and DOX, which resulted in a dual-color fluorescence turn-on. As shown in Fig. 6B, the blue fluorescence of TPE increased gradually in lysosomes as the drug is activated, while the increased red fluorescence in the nucleus confirmed the subcellular distribution of DOX. As TPE and DOX showed distinct fluorescent colors, by studying the spatiotemporal pattern of TPE and DOX through fluorescence imaging, one can easily understand the release time, drug-release site and the subcellular distribution of the drugs.

**Fig. 7:**
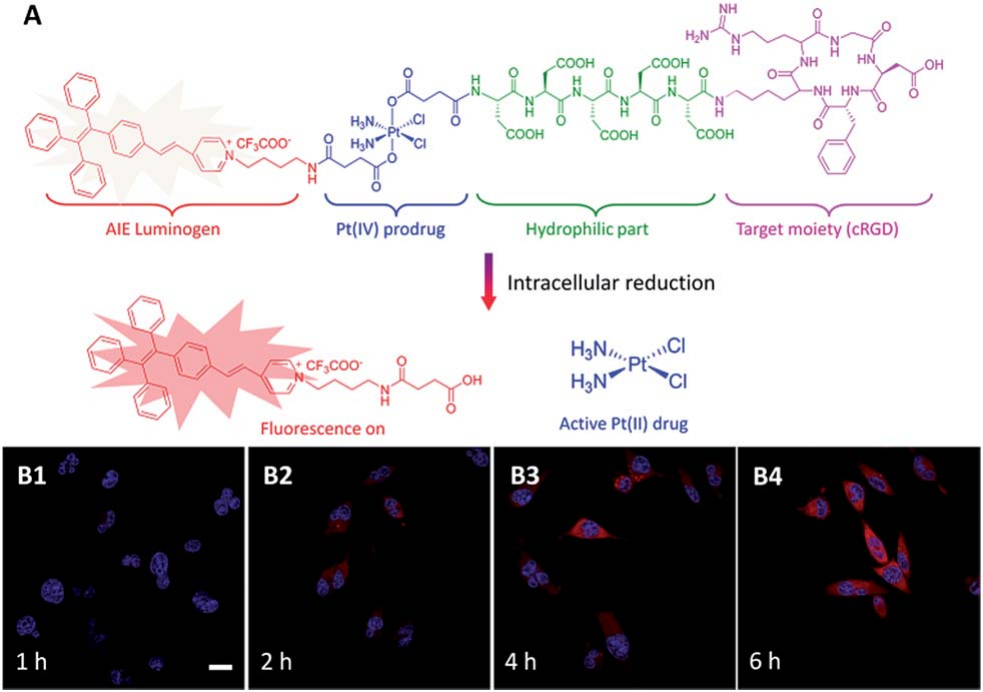
(A) Schematic illustration of the TPE-based prodrug design strategy and (B) the fluorescence turn-on monitoring of drug activation with different incubation times. Reprinted with permission from ref. 35. Copyright 2014 Royal Society of Chemistry.

Apart from DOX, cisplatin is another chemo-drug which has been extensively used in clinics to treat a broad spectrum of human cancers, but it suffers from severe side effects which limit its clinical use. To overcome these limitations, its nontoxic form as Pt(iv) complexes are usually used as a prodrug which can be intracellularly reduced to the active Pt(ii) complex to restore their latent cytotoxic activity.^34^ To capture the dynamic Pt(iv) prodrug activation, Liu and Tang *et al.* synthesized and evaluated a targeted theranostic platinum(iv) prodrug delivery system (Fig. 7).^35^ Cyclic arginine–glycine–aspartic acid (cRGD) and five units of aspartic acid (D) were inserted between Pt(iv) and the AIEgens to endow the probe with targeting ability and good water-solubility.^36,37^ As shown in Fig. 7B1–4, the probe is practically non-emissive in aqueous media and can selectively enter integrin overexpressed MDA-MB-231 cells. Pt(iv) was reduced intracellularly to yield the toxic Pt(ii) drug and generated a highly emissive AIE residue. The light-up fluorescence provides a reliable signal for quantitative analysis of the active drug concentration with high signal-to-noise ratio. As expected, the prodrug shows more potent cancer cell inhibition to MDA-MB-231 cells as compared with MCF-7 cells with low integrin expression.

Later, the same research group further designed and constructed a traceable targeted theranostic DDS containing two prodrugs for dual-drug tracking with synergistic anticancer effects.^38^ In this work, the two axial positions of the Pt(iv) prodrug was functionalized with blue-emissive TPE as an energy donor and a red emissive anticancer drug DOX as the energy acceptor. Before drug activation, the fluorescence of TPE is quenched due to energy transfer to DOX, while red fluorescence of DOX upon direct excitation at 488 nm can be utilized for the prodrug intracellular distribution monitoring. After cellular uptake, the prodrug was first located in endo/lysosome with red fluorescence. Concomitant with the drug activation, the separation between TPE and DOX resulted in fluorescence recovery of the blue emissive TPE and its fluorescence was intensified within 2 h, indicative of drug activation for both Pt(ii) and DOX. This is further evidenced by the red fluorescence of DOX observed at 6 h in the nuclei. The simultaneous DOX and cisplatin activation resulted in synergistic anticancer effects. The IC_50_ of cRGD–TPE–Pt–DOX against MDA-MB-231 cells is 0.69 mM, which is 2.3 and 26.6 times lower than those of DOX (IC_50_ = 1.6 mM) and cisplatin (IC_50_ = 18.1 mM) treatments, respectively. Such a dual-acting theranostic DDS with prodrug tracking, real-time drug activation monitoring and synergistic anticancer effects is beneficial for cancer therapy.

**Fig. 8:**
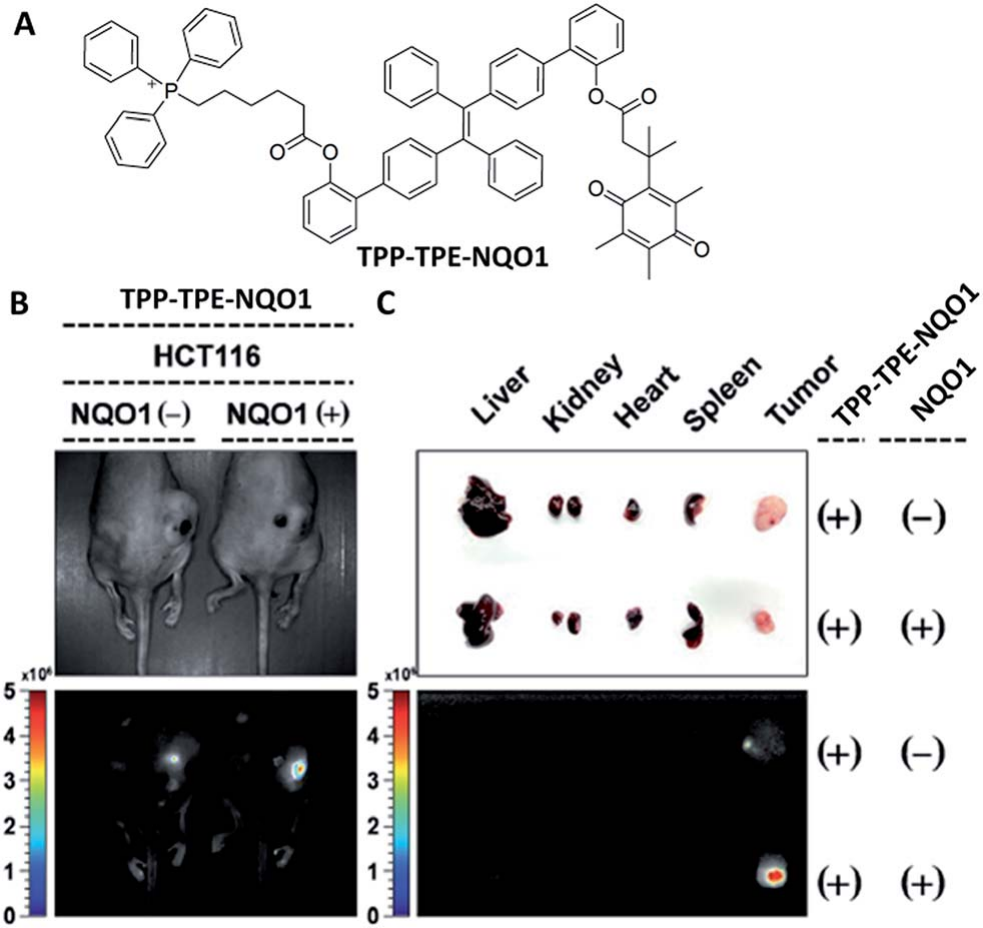
(A) Chemical structure of TPP–TPE–NQO1. (B) Representative images of mice bearing HCT116 cells, pretreated with control plasmid (left) and NQO1 expression plasmid (right) under white light (top) and the representative *in vivo* fluorescence images of TPP–TPE–NQO1 accumulation in xenograft tumors (bottom; *E*_x_: 430–480 nm, *E*_m_: 490–550 nm). (C) Dissected organs and tumors (top) and their fluorescence images (bottom) of TPP–TPE–NQO1-injected mice (*E*_x_: 430–480 nm, *E*_m_: 490–550 nm). Reprinted with permission from ref. 39. Copyright 2016 Royal Society of Chemistry.

DDSs that target sub-organelle combined with tumor microenvironment associated enzymatic drug activation can enhance the selectivity of cancer therapy. The mitochondrion is one of the most vital subcellular organelles which is known as the powerhouse of both normal and malignant cells. The combination of microenvironment associated enzymatic drug activation and the subsequent mitochondria targeting can induce the disruption of major metabolic pathways in mitochondria to lead to effective cancer cell death.^39^ Kim *et al.* developed a mitochondria targeted prodrug TPP–TPE–NQO1, which is composed of a TPE derivative conjugated with an NAD(P)H:quinone oxidoreductase-1 (NQ-1) cleavable masking unit with a mitochondria targeting triphenylphosphine (TPP) moiety. The non-fluorescent TPP–TPE–NQO1 showed preferential accumulation in cancer cells and underwent rapid reduction of the quinone scaffold in the presence of NQ-1, resulting in cyclisation, which releases the active form of the TPE derivative synchronously with strong blue fluorescence turn-on. The released AIEgen can specifically target mitochondria and induce the disruption of major metabolic pathways.^40^ As shown in Fig. 8, the probe specifically accumulated in the tumor tissue, and drug activation was only observed in the case of NQO1 positive cells which were clearly demonstrated by the strong blue fluorescence as compared with the control both in *in vivo* and *ex vivo* studies. The work confirmed that the therapeutic effect was dependent on both the sub-organelle localization and specific *in situ* prodrug activation.

**Fig. 9:**
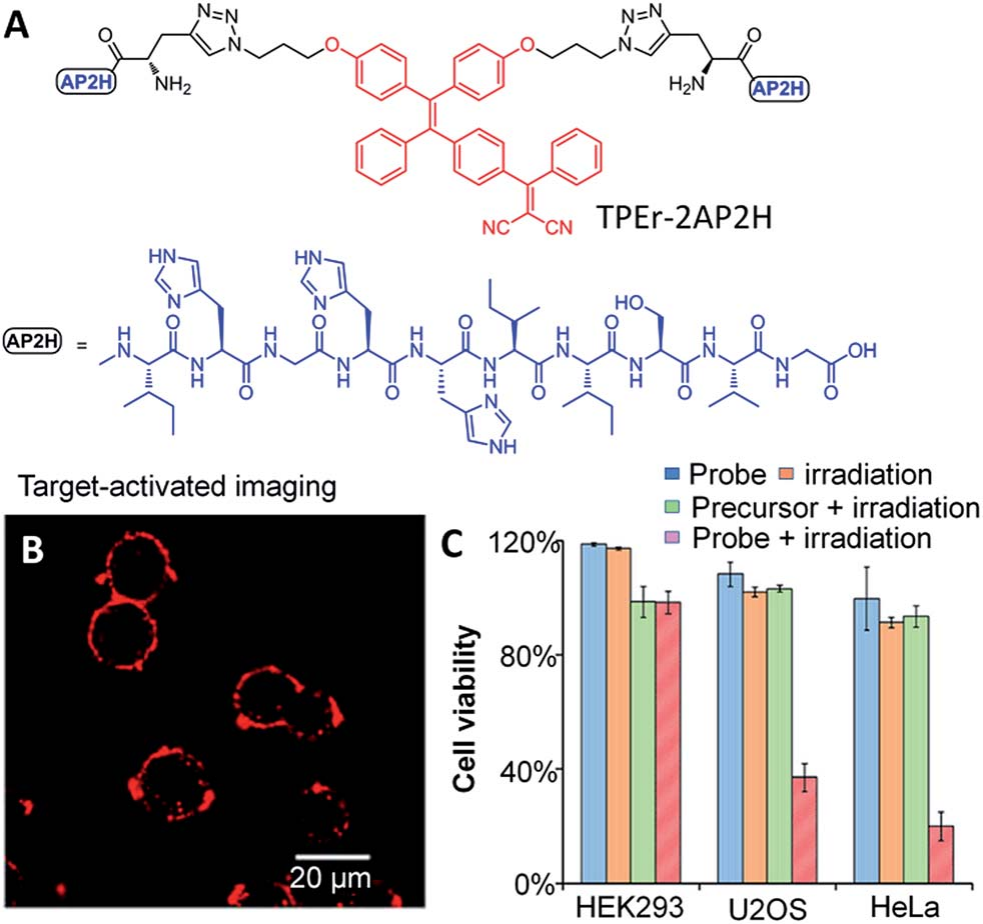
(A) Chemical structure of a red emissive AIE probe for targeted bioimaging and photodynamic therapy of cancer cells. (B) CLSM images of HeLa cells after incubation with the probe (10 μM) in an acidic (pH 5.5) environment. The probe was excited with a 488 nm laser, and the emission was collected with a 580–680 nm filter. (C) Comparison of cell viability for HeLa cells, U2OS cells, and HEK293 cells under different conditions. Reprinted with permission from ref. 48. Copyright 2014 American Chemical Society.

Apart from the various therapeutic drugs for cancer therapy, photosensitizer (PS) drugs as an emerging therapeutic agent have received increasing attention as photodynamic therapy (PDT) can be spatiotemporally controlled with light irradiation.^41^ PDT requires a PS to generate cytotoxic reactive oxygen species (ROS) upon light irradiation to induce cell death. Recently, some AIEgens were engineered to show therapeutic effects.^42–47^ Unlike conventional porphyrin based PSs, AIEgen PSs do not suffer from quenched fluorescence or obviously reduced photosensitizing efficiency in the aggregate state. Recently, Zhang and Yang *et al.* developed a red emissive probe TPEr–2AP2H containing a TPE derived PS and two peptides that could specifically target tumor overexpressed biomarker LAPTM4B proteins (Fig. 9A).^48^ The introduction of a [PhC=C(CN)_2_] moiety to the TPE core endowed the AIEgens with photosensitivity for ROS generation. As shown in Fig. 9B, the red fluorescence of the AIEgen TPEr can be turned-on upon binding between AP2H (IHGHHIISVG) peptides and the LAPTM4B proteins. This endows the probe with cancer cell specific uptake and *in situ* fluorescence turn-on to monitor the PS activation. The selective cancer cell ablation with fluorescence-guided light irradiation is shown in Fig. 9C, which reveals effective cancer cell (HeLa and U2OS) ablation with minimum toxicity to HEK293 normal cells. Meanwhile, Liu *et al.* developed a dual targeted theranostic probe based on TPECM with activatable PDT for targeted cancer cell ablation.^49^ The probe contains a cRGD peptide to target integrins overexpressed on the cancer cell surface with a second peptide sequence to respond to cathepsin B enzymes overexpressed in cancer cell lysosomes. The probe is non-fluorescent by itself. Once it is trapped in a lysosome, cathepsin B cleaves the probe, and releases the hydrophobic TPECM residues to generate red fluorescence. This indicated the activation of the photosensitization properties of TPECM, which was subsequently used for image-guided PDT. The dual-targeted design strategy enables light-up cancer-cell imaging with a high signal-to-noise ratio and led to highly selective ablation of MDA-MB-231 cancer cells with minimum toxicity to 293T normal cells under the same experimental conditions. This probe design offers a good opportunity to develop activatable PSs without incorporating any quencher or energy acceptor, which can also achieve enhanced fluorescence and phototoxicity in the aggregate state upon activation by tumor related stimuli.

**Fig. 10:**
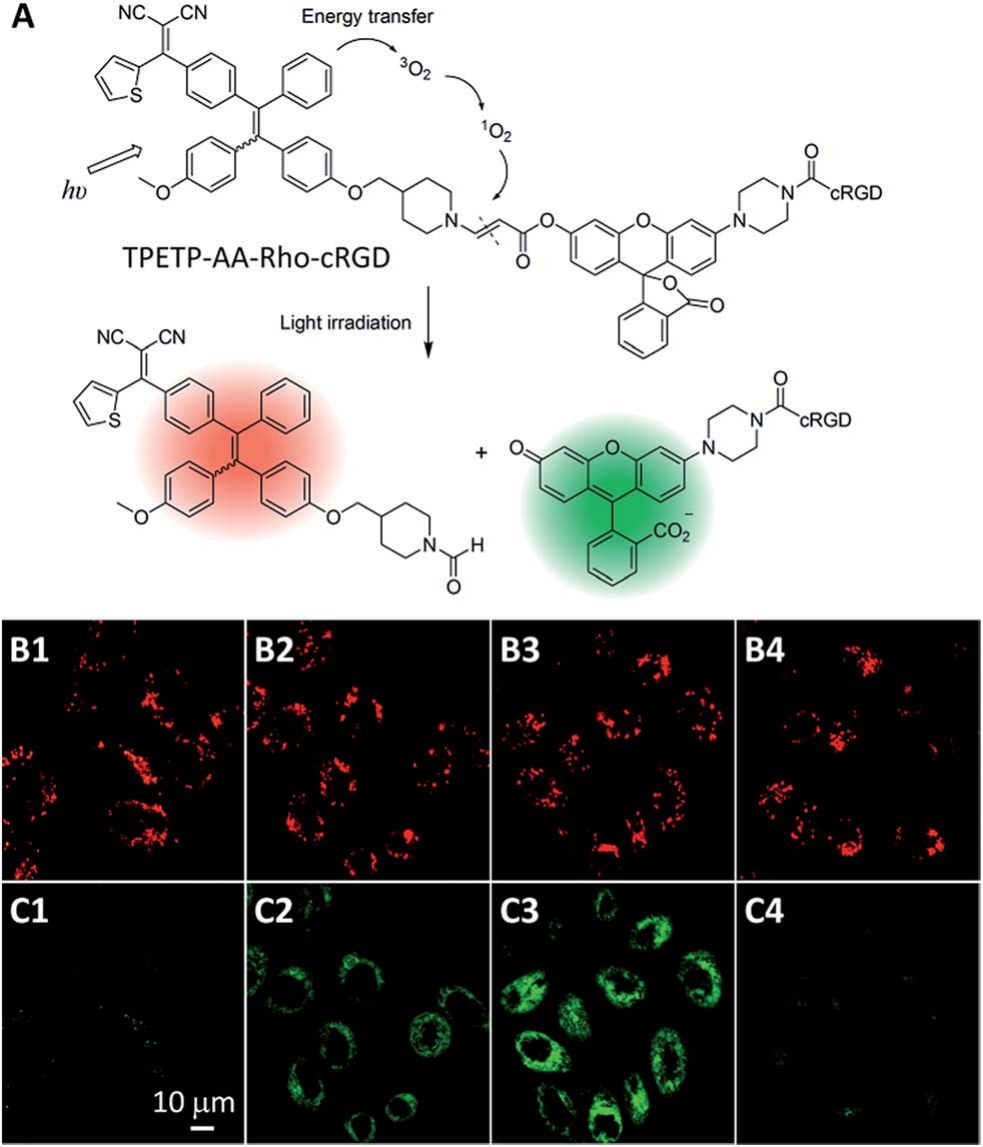
(A) Structure of theranostic probe TPETP–AA–Rho–cRGD and schematic representation of the proposed singlet oxygen self-reporting mechanism. (B and C) CLSM images of the probe (10 μM), incubated MDA-MB-231 cells, with light irradiation at a power density of 0.10 W cm^–2^ for (B1 and C1) 0 min, (B2 and C2) 2 min, (B3 and C3) 4 min and (B4 and C4) 4 min in the presence of Asc (100 μM). (B1–B4) Red fluorescence (TPETP, *E*_x_: 405 nm; *E*_m_: > 650 nm); (C1–C4) green fluorescence (Rho, *E*_x_: 488 nm, *E*_m_: 505–525 nm). Reprinted with permission from ref. 51. Copyright 2015 Royal Society of Chemistry.

As the mechanism of PDT is based on PSs that can react with oxygen under light irradiation to generate ROS, the amount of ROS that can be generated from PSs upon light irradiation is crucial to the therapeutic outcome. Although several fluorescence turn-on probes have been developed for monitoring ROS generation during PDT,^50^ it remains a challenge to *in situ* monitor ROS generation in living systems due to the extremely small radius of action (<20 nm) and short lifetime (<40 ns) of ROS.^51^ To tackle this issue, Liu *et al.* developed a probe which is composed of an AIEgen photosensitizer conjugated with a fluorogenic green emissive rhodol dye through a ROS cleavable aminoacrylate linker (Fig. 10A).^52^ Before white light irradiation, the probe can self-assemble into nanoaggregates with red fluorescence of the TPETP, which does not change upon white light irradiation. The red signal can thus be used for PS self-tracking (Fig. 10B). In contrast, as shown in Fig. 10C, the green fluorescence of rhodol increased gradually upon light irradiation, which should be due to the generated ROS that can cleave the aminoacrylate linker, leading to the formation of highly green emissive fluorophore 6-hydroxy-3*H*-xanthen-3-one. This green fluorescence turn-on was greatly inhibited in the presence of ROS scavenger vitamin C, further confirming the ROS induced green fluorescence change. This design strategy provides a facile method to *in situ* and real-time monitor PS drug activation during photodynamic ablation of cancer cells.

## Therapeutic response monitoring

Although the above probes can monitor drug distribution and drug activation in cancer cells, they do not offer any information on the drug therapeutic responses. To monitor the therapeutic responses of a specific DDS is of high importance for clinical applications as it can guide the therapeutic decisions to evaluate the therapeutic regimes. The current clinical method to evaluate the therapeutic effect of cancer treatment is to measure the tumor size change using a magnetic resonance imaging (MRI) study.^53^ However, this method is unsatisfactory in the early evaluation of the therapeutic effects as the tumor size change is not obvious at the early stage of therapy. As a consequence, DDSs that can simultaneously deliver the anticancer drugs and offer real-time monitoring of the therapeutic responses *in situ* are highly desirable. Recently, by incorporation of apoptosis sensors or apoptotic markers (*e.g.* ethidium) into prodrugs or nanoparticles, *in situ* monitoring of the drug therapeutic effects has also been realized.^11,12^ In addition, some of the theranostic DDSs based on AIEgens have also been developed for real-time monitoring of the therapeutic responses which are discussed in this section.^54–56^

Apoptosis, or programmed cell death, is an important and active regulatory pathway of cell growth and proliferation.^57^ It is known that most anticancer drugs kill cancer cells through an apoptosis pathway by activating caspase enzymes within a short period of time.^58^ Therefore, the accurate detection of caspase enzyme activation in cells could be a reliable strategy to predict the therapeutic response of drugs at a very early stage. Recently, we have developed several light-up fluorescent probes based on AIEgens for real-time monitoring of cell apoptosis *via* the detection of caspase enzyme activation.^59–61^ Although these probes can monitor drug induced cell apoptosis, they are not able to quantify the therapeutic responses of drugs in real-time and *in situ* as the distribution or subcellular location of drugs and probes may not be the same.

**Fig. 11:**
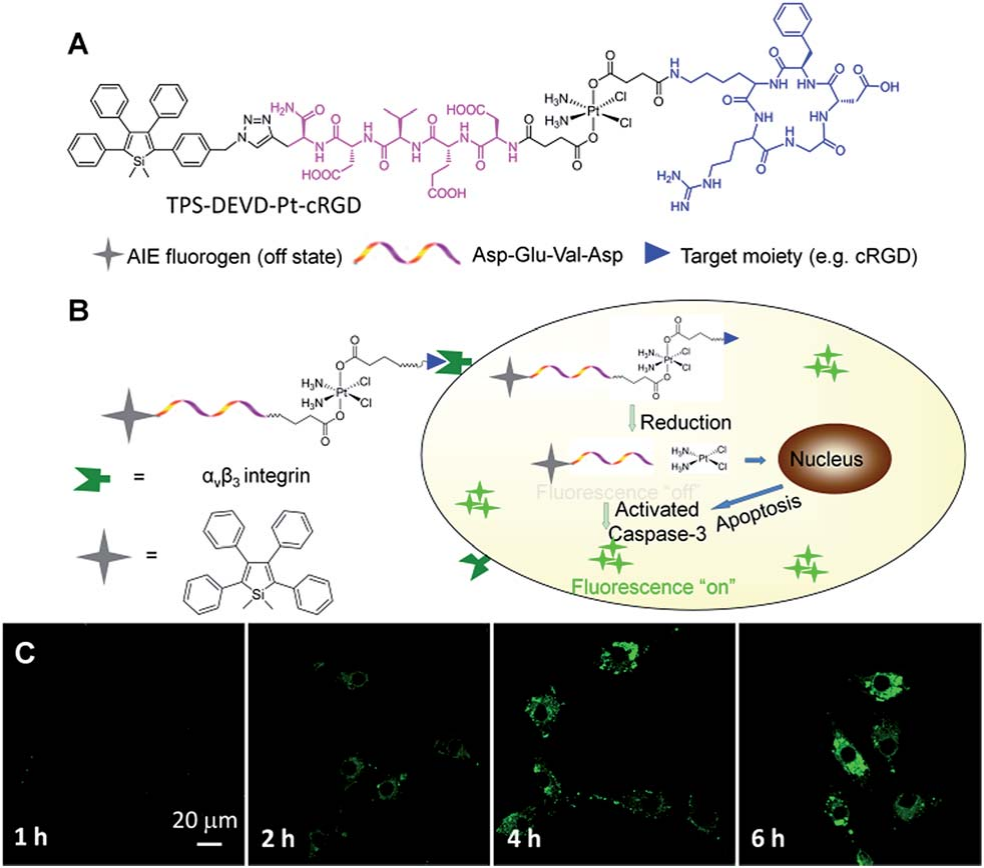
(A) The molecular structure of the targeted theranostic platinum(iv) prodrug TPS–DEVD–Pt–cRGD. (B) Schematic illustration of the targeted theranostic platinum(iv) prodrug TPS–DEVD–Pt–cRGD with a built-in AIE light-up apoptosis sensor for non-invasive *in situ* early evaluation of its therapeutic responses. (C) Real-time CLSM images displaying the apoptotic progress of TPS–DEVD–Pt–cRGD (5 μM) stained U87-MG cells. The green fluorescence is from TPS (*E*_x_: 488 nm, *E*_m_: 505–525 nm). Reprinted with permission from ref. 54. Copyright 2014 American Chemical Society.

To solve this problem, Liu and Tang *et al.* developed a targeted theranostic platinum(iv) prodrug delivery system TPS–DEVD–Pt–cRGD for drug delivery to targeted cancer cells and simultaneous non-invasive early evaluation of the therapeutic response of an activated drug *in situ* (Fig. 11).^54^ Cisplatin was modified to the nontoxic form of the platinum(iv) prodrug and its two axial positions were functionalized with a targeting peptide cRGD and a built-in AIE light-up apoptosis sensor TPS–DEVD, which is composed of tetraphenylsilole (TPS) and a caspase-3 enzyme specific Asp-Glu-Val-Asp (DEVD) peptide. After cellular uptake by α_v_β_3_ integrin overexpressed cancer cells, the Pt(iv) prodrug was reduced to form an active Pt(ii) drug and simultaneously release TPS–DEVD. The toxic Pt(ii) further induces the cell apoptosis and activates caspase-3 enzyme, which can be specifically detected by TPS–DEVD with TPS fluorescence light-up. Fig. 11C shows the time dependent confocal images of prodrug incubated U87-MG cells, showing progressive light-up of the cells which are revealed by the gradual increase of the green fluorescence of TPS. The apoptosis-induced fluorescence change in the prodrug incubated U87-MG cells exhibits a good correlation with the cell viability, confirming that the prodrug delivery system is useful for drug delivery and simultaneous early assessment of the drug therapeutic efficacy, which is essential to guide therapeutic decisions.

**Fig. 12:**
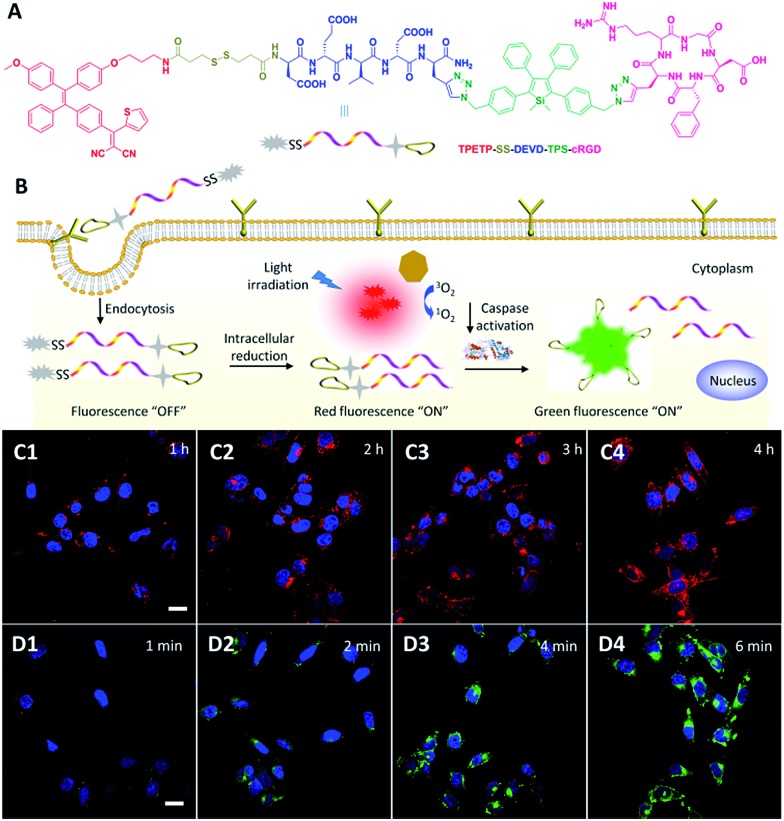
(A) Chemical structure of theranostic DDS TPETP–SS–DEVD–TPS–cRGD. (B) Schematic illustration of the dual-targeted probe for real-time and *in situ* monitoring of PS activation and therapeutic responses. (C) Confocal images of MDA-MB-231 cells after incubation with the probe for different times. The blue fluorescence is from the cell nuclei stained with Hoechst (*E*_x_: 405 nm; *E*_m_: 430–470 nm); the red fluorescence is from the AIE residue (*E*_x_: 405 nm; *E*_m_: > 560 nm). (D) Confocal images of MDA-MB-231 cells upon incubation with the probe for 4 h with light irradiation for different times. The blue fluorescence is from the nuclei of the cells stained with Hoechst; the green fluorescence is from the TPS residue (*E*_x_: 405 nm; *E*_m_: 505–525 nm). Reprinted with permission from ref. 55. Copyright 2015 Wiley-VCH.

To visualize drug activation and *in situ* monitoring of the therapeutic response, the same group further developed a multifunctional theranostic DDS, which is composed of an AIEgen photosensitizer with red-emission and a built-in green emissive activatable AIE light-up apoptosis sensor for targeted PDT and real-time monitoring of drug activation and prediction of the therapeutic response (Fig. 12).^55^ The non-emissive probe can be selectively taken-up by cancer cells with α_v_β_3_ integrin overexpression. As shown in Fig. 12C, after entering the cancer cells, the abundant intracellular glutathione (GSH) cleaves the disulfide group of the DDS to activate the TPETP with red fluorescence turn-on and simultaneously releases the apoptosis sensor. The released apoptosis sensor remains non-emissive due to the hydrophilicity of the DEVD substrate. Under light illumination, as shown in Fig. 12D, the green fluorescence of the TPS residue in the apoptosis sensor is turned on through enzymatic reaction of the activated caspase enzymes in the cell apoptosis process induced by the generated ROS, which can be used for the monitoring of the therapeutic response. This multifunctional probe with sequentially red and green fluorescence turn-on can simultaneously monitor the drug activation and provide early evaluation of the therapeutic response which is expected to provide a clearer picture of drug delivery and cancer therapy.

**Fig. 13:**
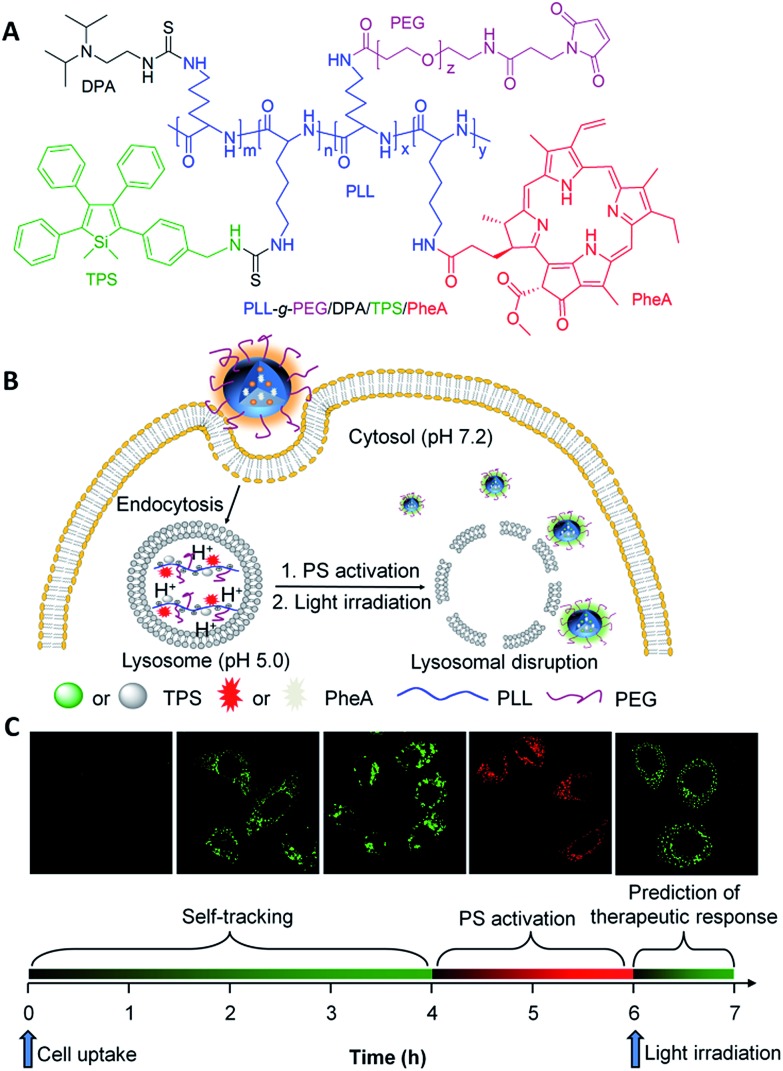
(A) Chemical structure of theranostic DDS PLL-*g*-PEG/DPA/TPS/PheA. (B and C) Schematic illustration of PLL-*g*-PEG/DPA/TPS/PheA used for self-tracking, cancer cell imaging, phototoxicity restoration (PS activation) in the acidic lysosome, and *in situ* monitoring of lysosomal membrane disruption as an indicator of therapeutic responses and cell death prediction. Reprinted with permission from ref. 56. Copyright 2015 Wiley-VCH.

Despite the great efforts that have been made toward theranostic DDSs to visualize drug delivery processes, most of them can only monitor one or two of the processes.^8^ As a successful example of reporting both the drug distribution and therapeutic function, Yang *et al.* incorporated lysotracker and a porphyrin PS into a NP. The biodistribution of the PS and the ROS triggered lysosomal destruction were monitored with red and NIR emitters, respectively.^62^ It remains a challenge to develop a single theranostic DDS that is able to simultaneously monitor the drug distribution, drug activation and predict the therapeutic response. Recently, Liu and Tang *et al.* developed such a theranostic DDS utilizing the exact opposite photophysical properties of ACQ PSs and AIEgens. A pH-responsive polymeric nanoprobe was developed, which contains a red emissive PS (pheophorbide A, PheA) with ACQ properties and a green emissive TPS with AIE characteristics for tracing the whole process of PDT (Fig. 13).^56^ As shown in Fig. 13B and C, under physiological conditions (pH 7.4), the probe self-assembles into NPs, which shows a low phototoxicity of PheA with weak red fluorescence, and strong green fluorescence of TPS for monitoring the distribution of PSs. After being internalized by cancer cells through endocytic pathways and entrapped in endo/lysosomes (pH 5.0), the nanoprobe disassembles to the molecular state due to the protonation of diisopropylamino (DPA) side groups, yielding a strong red fluorescence of PheA with restored phototoxicity for PS activation monitoring. Meanwhile, the green fluorescence of TPS is weakened due to the free rotation of phenyl rings in the molecular state. Under light illumination, the generated ROS disrupted the lysosomal membrane and triggered cell apoptosis. The probe leaked into the cytoplasm (pH 7.2), where it re-assembled into NPs with a green TPS fluorescence restored for evaluation of the therapeutic response *in situ*. The green fluorescence of TPS in the endo/lysosome can be used for biodistribution monitoring while the fluorescence located in the cytoplasm can be used for the prediction of therapeutic response. This nanoprobe design represents a novel strategy for multiple purpose imaging and therapy to visualize the different processes of drug delivery and cancer therapy.

## Conclusions

Theranostic DDSs are one of the most rapidly growing fields in personalized cancer therapy, which have had marked impacts on simultaneous drug delivery and cancer diagnosis. The theranostic DDSs, which typically consist of imaging agents and drugs conjugated through cleavable linkers with the modification of targeting units, can operate intracellularly through activation by various stimulators such as GSH, pH or tumor specific enzymes.^3^ The unique properties of AIEgens simplified the development of light-up probes without the incorporation of energy acceptor or quencher moieties. In this mini-review, we presented general design strategies and discussed typical examples of theranostic DDSs based on AIEgens for real-time monitoring of the drug distribution, drug activation and early evaluation of therapeutic responses. Thanks to their unique photophysical properties, which are opposite to those of the traditional fluorescent dyes, AIEgens show additional advantages in the development of light-up probes without the incorporation of any quencher moieties, which simplified the design of multifunctional theranostic DDSs to visualize all of the processes of drug delivery. The direct use of AIEgen PSs as therapeutic agents further improved the convenience of developing theranostic DDSs with an extremely simple design strategy to visualize the drug delivery processes.

Future work of formulating advanced theranostic DDSs based on AIEgens should be focused on, addressing the following issues. Firstly, the current AIEgen based light-up drug delivery probes largely require good water-solubility to ensure a low background signal, which is difficult to achieve for hydrophobic drugs, such as DOX or paclitaxel (PTX). This calls for the development of next generation AIEgens with fluorogenic properties, such as those with excited state intramolecular proton transfer (ESIPT) properties to bypass the background issues associated with the probe solubility. Secondly, the theranostic DDSs covered in this review mainly used AIEgens with absorption in the blue and green regions, which are not very suitable for *in vivo* applications due to the limited tissue penetration depth and strong autofluorescence in the visible region. One option is to design AIEgens with longer absorption wavelengths or multiphoton absorption with desirable functionalities. The other option is to use upconversion nanoparticles to convert NIR light to short wavelength light to excite AIEgens. With the advancement of both materials and biology, we anticipate that the integrated theranostic DDSs that can be further simplified to incorporate more functions will provide more important information on cancer therapy to help realize the ultimate goal of personalized medicine in the near future.

## References

[cit1] Rosen H., Abribat T. (2005). Nat. Rev. Drug Discovery.

[cit2] Langer R. (1998). Nature.

[cit3] Tong R., Tang L., Ma L., Tu C., Baumgartner R., Cheng J. (2014). Chem. Soc. Rev..

[cit4] Yoo J. W., Irvine D. J., Discher D. E., Mitragotri S. (2011). Nat. Rev. Drug Discovery.

[cit5] Wang Y., Shim M. S., Levinson N. S., Sung H. W., Xia Y. (2014). Adv. Funct. Mater..

[cit6] Yuan Y., Liu J., Liu B. (2014). Angew. Chem., Int. Ed..

[cit7] Wang Y., Song S., Liu J., Liu D., Zhang H. (2015). Angew. Chem., Int. Ed..

[cit8] Kumar R., Shin W. S., Sunwoo K., Kim W. Y., Koo S., Bhuniya S., Kim J. S. (2015). Chem. Soc. Rev..

[cit9] Ye M., Wang X., Tang J., Guo Z., Shen Y., Tian H., Zhu W. (2016). Chem. Sci..

[cit10] Tian J., Zhou J., Shen Z., Ding L., Yu J., Ju H. (2015). Chem. Sci..

[cit11] Min Y., Li J., Liu F., Yeow E. K., Xing B. (2014). Angew. Chem., Int. Ed..

[cit12] Kumar R., Han J., Lim H. J., Ren W. X., Lim J. Y., Kim J. H., Kim J. S. (2014). J. Am. Chem. Soc..

[cit13] Ding D., Li K., Liu B., Tang B. Z. (2013). Acc. Chem. Res..

[cit14] Mei J., Leung N. L. C., Kwok R. T. K., Lam J. W. Y., Tang B. Z. (2015). Chem. Rev..

[cit15] Zhao Z., He B., Tang B. Z. (2015). Chem. Sci..

[cit16] Liang J., Tang B. Z., Liu B. (2015). Chem. Soc. Rev..

[cit17] Kwok R. T., Leung C. W., Lam J. W., Tang B. Z. (2015). Chem. Soc. Rev..

[cit18] Shao A., Xie Y., Zhu S., Guo Z., Guo J., Shi P., James T. D., Tian H., Zhu W. H. (2015). Angew. Chem., Int. Ed..

[cit19] Li K., Liu B. (2014). Chem. Soc. Rev..

[cit20] Lu H., Zheng Y., Zhao X., Wang L., Ma S., Han X., Xu B., Tian W., Gao H. (2016). Angew. Chem., Int. Ed..

[cit21] Escobedo J. O., Rusin O., Lim S., Strongin R. M. (2010). Curr. Opin. Chem. Biol..

[cit22] Birks J. B., Photophysics of Aromatic Molecules, Wiley, London, 1970.

[cit23] Oh E., Liu R., Nel A., Gemill K. B., Bilal M., Cohen Y., Medintz I. L. (2016). Nat. Nanotechnol..

[cit24] Zhang C., Jin S., Li S., Xue X., Liu J., Huang Y., Jiang Y., Chen W. Q., Zou G., Liang X. J. (2014). ACS Appl. Mater. Interfaces.

[cit25] Guan W., Zhou W., Lu C., Tang B. Z. (2015). Angew. Chem., Int. Ed..

[cit26] Wang H., Liu G., Gao H., Wang Y. (2015). Polym. Chem..

[cit27] Wang H., Liu G., Dong S., Xiong J., Du Z., Cheng X. (2015). J. Mater. Chem. B.

[cit28] Yuan Y., Xu S., Zhang C.-J., Liu B. (2016). Polym. Chem..

[cit29] Xue X., Zhao Y., Dai L., Zhang X., Hao X., Zhang C., Huo S., Liu J., Liu C., Kumar A., Chen W.-Q., Zou G., Liang X.-J. (2014). Adv. Mater..

[cit30] Yuan Y., Zhang C., Liu B. (2015). Angew. Chem., Int. Ed..

[cit31] Jin G., Feng G., Qin W., Tang B., Liu B., Li K. (2016). Chem. Commun..

[cit32] Rautio J., Kumpulainen H., Heimbach T., Oliyai R., Oh D., Jarvinen T., Savolainen J. (2008). Nat. Rev. Drug Discovery.

[cit33] Xue X., Jin S., Zhang C., Yang K., Huo S., Chen F., Zou G., Liang X. J. (2015). ACS Nano.

[cit34] Graf N., Lippard S. J. (2012). Adv. Drug Delivery Rev..

[cit35] Yuan Y., Chen Y., Tang B. Z., Liu B. (2014). Chem. Commun..

[cit36] Yuan Y., Zhang C. J., Liu B. (2015). Chem. Commun..

[cit37] Yuan Y., Kwok R. T., Feng G., Liang J., Geng J., Tang B. Z., Liu B. (2014). Chem. Commun..

[cit38] Yuan Y., Kwok R. T., Zhang R., Tang B. Z., Liu B. (2014). Chem. Commun..

[cit39] Wallace D. C. (2012). Nat. Rev. Cancer.

[cit40] Shin W., Lee M., Verwilst P., Lee J., Chi S., Kim J. S. (2016). Chem. Sci..

[cit41] Cheng L., Wang C., Feng L., Yang K., Liu Z. (2014). Chem. Rev..

[cit42] Yuan Y., Feng G., Qin W., Tang B. Z., Liu B. (2014). Chem. Commun..

[cit43] Hu Q., Gao M., Feng G., Liu B. (2014). Angew. Chem., Int. Ed..

[cit44] Yuan Y., Xu S., Cheng X., Cai X., Liu B. (2016). Angew. Chem., Int. Ed..

[cit45] Xu S., Yuan Y., Cai X., Zhang C., Hu F., Liang J., Zhang G., Zhang D., Liu B. (2015). Chem. Sci..

[cit46] Zhang C., Hu Q., Feng G., Zhang R., Yuan Y., Liu B. (2015). Chem. Sci..

[cit47] Huang Y., Zhang G., Hu F., Jin Y., Zhao R., Zhang D. (2016). Chem. Sci..

[cit48] Hu F., Huang Y., Zhang G., Zhao R., Yang H., Zhang D. (2014). Anal. Chem..

[cit49] Yuan Y., Zhang C. J., Gao M., Zhang R., Tang B. Z., Liu B. (2015). Angew. Chem., Int. Ed..

[cit50] Kim S., Tachikawa T., Fujitsuka M., Majima T. (2014). J. Am. Chem. Soc..

[cit51] Ogilby P. R. (2010). Chem. Soc. Rev..

[cit52] Yuan Y., Zhang C., Xu S., Liu B. (2016). Chem. Sci..

[cit53] Fass L. (2008). Mol. Oncol..

[cit54] Yuan Y., Kwok R. T., Tang B. Z., Liu B. (2014). J. Am. Chem. Soc..

[cit55] Yuan Y., Zhang C., Kwok R. T. K., Xu S., Zhang R., Wu J., Tang B., Liu B. (2015). Adv. Funct. Mater..

[cit56] Yuan Y., Kwok R. T., Tang B. Z., Liu B. (2015). Small.

[cit57] Vaux D. L., Korsmeyer S. J. (1999). Cell.

[cit58] Olsson M., Zhivotovsky B. (2011). Cell Death Differ..

[cit59] Yuan Y., Zhang R., Cheng X., Xu S., Liu B. (2016). Chem. Sci..

[cit60] Shi H., Kwok R. T. K., Liu J., Xing B., Tang B. Z., Liu B. (2012). J. Am. Chem. Soc..

[cit61] Yuan Y., Zhang C., Ryan T. K. K., Mao D., Tang B. Z., Liu B. (2017). Chem. Sci..

[cit62] Tian J., Ding L., Ju H., Yang Y., Li X., Shen Z., Zhu Z., Yu J. S., Yang C. J. (2014). Angew. Chem., Int. Ed..

